# An Innovative Platform Merging Elemental Analysis and Ftir Imaging for Breast Tissue Analysis

**DOI:** 10.1038/s41598-019-46056-4

**Published:** 2019-07-08

**Authors:** Mohamed H. M. Ali, Fazle Rakib, Khalid Al-Saad, Rafif Al-Saady, Erik Goormaghtigh

**Affiliations:** 1 Diabetes Research Center, Qatar Biomedical Research Institute (QBRI), Hamad Bin Khalifa University (HBKU), Qatar Foundation (QF), PO Box 34110, Doha, Qatar; 2 Department of Chemistry and Earth Sciences, Qatar University, Doha, Qatar; 3 Pathology and Laboratory Medicine, Al Ahli Hospital, Doha, Qatar; 4 Center for Structural Biology and Bioinformatics, Laboratory for the Structure and Function of Biological Membranes, Campus Plaine CP206/02, Université Libre de Bruxelles CP206/2, B1050 Brussels, Belgium

**Keywords:** Cancer, Oncology, Cancer, Oncology

## Abstract

Histopathology and immunohistology remain the gold standard for breast cancer diagnostic. Yet, these approaches do not usually provide a sufficiently detailed characterization of the pathology. The purpose of this work is to demonstrate for the first time that elemental analysis and Fourier transform infrared spectroscopy microscopic examination of breast tissue sections can be merged into one dataset to provide a single set of markers based on both organic molecules and inorganic trace elements. For illustrating the method, 6 mammary tissue sections were used. Fourier transform infrared (FTIR) spectroscopy images reported a fingerprint of the organic molecules present in the tissue section and laser ablation elemental analysis (LA-ICP-MS) images brought inorganic element profiles. The 6 tissue sections provided 31 10^6^ and 150,000 spectra for FTIR and LA-ICP-MS spectra respectively. The results bring the proof of concept that breast tissue can be analyzed simultaneously by FTIR spectroscopy and laser ablation elemental analysis (LA-ICP-MS) to provide in both case reasonably high resolution images. We show how to bring the images obtained by the two methods to a same spatial resolution and how to use image registration to analyze the data originating from both techniques as one block of data. We finally demonstrates the elemental analysis is orthogonal to all FTIR markers as no significant correlation is found between FTIR and LA-ICP-MS data. Combining FTIR and LA-ICP-MS imaging becomes possible, providing two orthogonal methods which can bring an unprecedented diversity of information on the tissue. This opens a new avenue of tissue section analyses providing unprecedented diagnostic potential.

## Introduction

While in the US the incidence of breast cancer has been growing moderately in recent years, in the Gulf Cooperation Council Countries it grew by 40% in the last 12 years^1^. In clinical practice, the analysis of tissue samples relies on the examination of microscopic structures observed in stained tissue sections. Robustness of that practice is limited as evidenced by inter- and intra-observer discrepancies. Staining specificity can be improved by immunostaining of a few key markers such as oestrogen and progesterone receptors, HER2, Ki-67 and some more^2^. In practice, the information obtained is very limited and definitively not sufficient to deliver accurate diagnostic, provide adequate therapy and result in satisfactory prognosis at individual level. Furthermore, tumors are heterogeneous^3,4^ and their behavior strongly depends on their microenvironment^5,6^. The lack of molecular information available at cell level when observing tissue section results in incomplete overview of the patient pathology. While analysis of genetic materials at cell level is not a viable option, some spectroscopic approaches accurately reflect the molecular content of the cells. Vibrational histopathology relies on FTIR or Raman imaging. It allows the discrimination of very closely related cell lines by providing, for each pixel of the tissue section image, full vibrational spectra which precisely report the biochemical content of the cells^7,8^. Fourier transform infrared (FTIR) spectroscopy in particular has shown its ability to recognize unique cancer features in the field of breast cancer^9–12^. A recent comparison of a series of breast cancer cell lines grown in 2D and 3D cultures by transcriptomic analysis and by FTIR imaging indicated that FTIR and transcriptomics are as sensitive to detect differences between cell lines and differences within cell lines induced by growing in a 3D environment instead of the regular 2D culture condition^13^. In turn, normal and tumor tissue in breast^7,14–17^, colon^7,18–20^, lung^7,21^, prostate^7,22–25^ and cervix^7,26–28^ can be distinguished using FTIR spectroscopy. The use of 2D correlation analysis within the FTIR dataset of breast cancer tissues indicates that a very significant number of FTIR contribution are cross-correlated, decreasing the number of independent potential markers in the spectra, which suggests the addition of biomarkers from other sources could be beneficial^29^. While FTIR relies on the organic molecules present in the tissue sections, laser ablation inductively coupled plasma mass spectrometry (LA-ICP-MS) is a complementary technique which provides elemental analysis mapped on a micrometer scale in thin sections of a soft tissue for up to 10–15 different essential elements. LA-ICP-MS enables identification and discrimination of elemental differences with an accuracy in the range of the part per billion (ppb). The sample is volatilized in an ablation chamber by a powerful ultraviolet beam. The resulting aerosol is then driven to the inductively coupled plasma device that fully decomposes the volatilized sample into simple elements which are inonized. The ionized elements are finally analysed by mass spectrometry. LA-ICP-MS provides a unique means to detect levels of trace elements including Fe, Cu and Zn that may be related to cancer development in breast tissue. Metal distribution in a tissue has been shown to be predictive of cancer behavior, potentially because elements such as Zn parallel the overexpression of extracellular matrix metalloproteinases (MMPs), in particular of MMP-11 which is involved in the degradation of the extracellular matrix and tumor progression^30^. While numerous previous FTIR studies and a more limited number of LA-ICP-MS studies indicate a very good potential to obtain information of diagnostic value, combining FTIR imaging with LA-ICP-MS, two orthogonal methods bringing information on the organic molecule composition (FTIR) and abundance of simple inorganic elements (LA-ICP-MS) could therefore constitute a particularly powerful approach to decipher the subtle variations present in breast tissue. In a recent study, Anyz *et al*.^31^ developed a methodology using image registration to overlay H&E-stained tissue section images and LA-ICP-MS images reporting Zn and Cu concentrations in 10 melanoma sections. We demonstrate here the feasibility of this approach by processing and merging FTIR and LA-ICP-MS breast tissue image data. After image registration and pixel resizing, the two set of data could be combined and analyzed simultaneously. It must be noted that quantification of the improvement for diagnostic purposes is beyond the scope of the present communication.

## Methods

### Tissue sections

Six Formalin-Fixed Paraffin-Embedded (FFPE) breast tissue samples were obtained from the histopathology laboratory at Al-Ahli Hospital, Doha, Qatar. Experimental protocols were approved by Qatar University and Al-Ahli Hospital ethical committees. All methods were performed in accordance with the relevant guidelines and regulations of Qatar University, Al-Ahli Hospital, and Université Libre de Bruxelles. All the material were taken anonymously, a consent form from AL-Ahli Hospital was signed by all patients. As described in Verdonck *et al*.^10^ for each FFPE breast tissue sample, 3 adjacent tissue sections were cut using a microtome. Paraffin was removed by incubation in 2 successive xylene baths for 20 minutes. Tissue rehydration was achieved through 3 successive ethanol baths with a decreasing gradient of ethanol (100%, 90%, 70%) for 15 minutes and 2 milliQ water baths for 10 minutes. For one 5 µm thick tissue section, standard H&E staining was performed. This section was used as a reference. A second adjacent 5 µm thick section, used for FTIR imaging, was deposited on a Kevley Technologies MIR low-e microscope glass slide. These glass slides are covered by thin metal layers. The surface actually consists of several layers of tin oxide and silver and their reflective surface allows the recording of FTIR spectra in reflection mode, sometimes called transflection mode. The data were collected in transflection mode from sample regions of 350 × 350 µm^2^. One FTIR image (unit image or tile) resulted in 4,094 spectra. As described earlier, to cover larger areas an automatic tiling combined several FTIR tiles in order to obtain one large mosaic FTIR image^29^. A third 10 µm thick section was used for LA-ICP-MS imaging. As shown in Table 1, the FTIR data included a total of 31 10^6^ infrared spectra, i.e. a mean number of spectra per image of 5 10^6^. With larger pixels, the total number of LA-ICP-MS spectra was just above 150,000. The slides were submitted to two pathologist and only when the diagnosis was concordant between the two, the case was included in the study (i.e each pathologist made his/her diagnosis without knowing the other pathologist diagnosis).

**Table 1:** Characteristics of the images analyzed in this work. One FTIR tile corresponds to 4096 pixels or 4096 spectra.

Section #	type	FTIR image size (in tiles)	FTIR spectrum number	LA-ICP-MS image size (in pixels)	LA-ICP-MS spectrum number
1	fibroadenoma	31 × 47 = 1,457	**5**,**967**,**872**	110 × 119	**13**,**090**
2	fibrocystic	45 × 35 = 1,575	**6**,**451**,**200**	160 × 118	**18**,**880**
3	ductal hyperplasia	39 × 34 = 1,326	**5**,**431**,**296**	163 × 160	**26**,**080**
4	fibroadenoma	26 × 35 = 910	**3**,**727**,**360**	113 × 159	**17**,**967**
5	ductal hyperplasia	38 × 20 = 760	**3**,**112**,**960**	256 × 215	**55**,**040**
6	Intraductal papilloma	36 × 40 = 1,440	**5**,**898**,**240**	157 × 148	**23**,**236**

**Figure 1:**
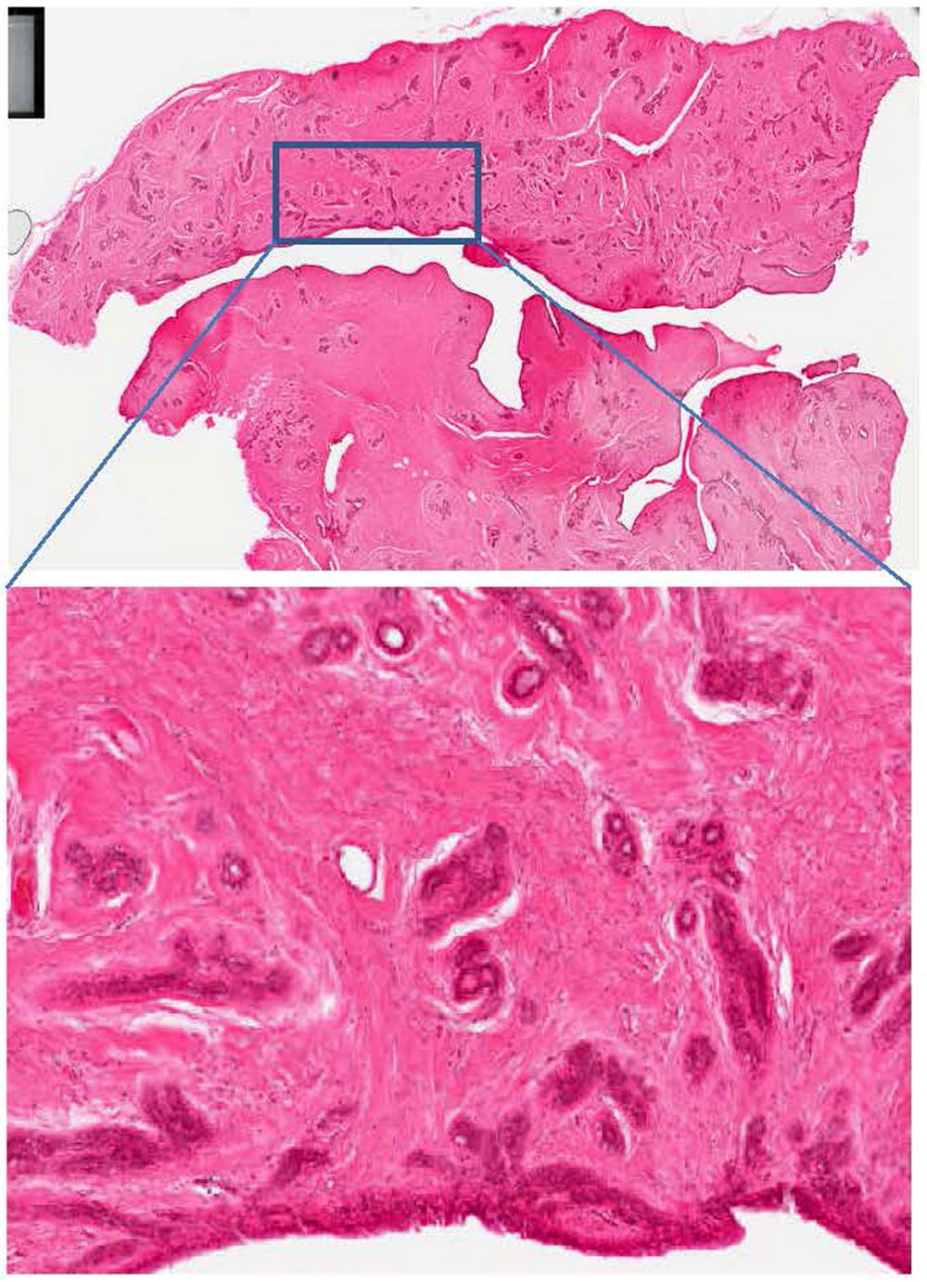
Image of an H&E stained fibroadenoma section described in the text. Bottom: enlargement of the area contained in the rectangle draw in the upper part of the figure (will be detailed in Fig. 2).

An example of tissue section is presented in Fig. 1 for a fibroadenoma. The section contains a piece of tissue showing loose fibroblastic stroma containing duct-like structures. These glandular or duct-like spaces are lined by single or multiple layers of cells that are regular with well-defined intact basement membrane.

### Imaging

FTIR spectroscopic images were obtained in transflection mode using an Agilent FTIR imaging microscope equipped with Mid-band MCT detector (12,000–600 cm^−1^, Mercury Cadmium Telluride). The images were obtained in the range of 4000–700 cm^−1^ with 128 scans per pixel, each pixel covering an area of 6.25 × 6.25 μm^2^ and 4 cm^−1^ spectral resolution.

As described elsewhere^32^, Laser Ablation Inductively Coupled Plasma Mass spectra were acquired using a laser ablation system (New Wave 213, ESI) equipped with a frequency quintupled neodymium-doped yttrium aluminium garnet (Nd:YAG) laser and a fast-washout ablation cell. The laser ablation device was coupled to a quadrupole ICP-MS system (iCAPQc, ThermoFisher Scientific, Bremen, Germany) using polytetrafluoroethylene tubing. Helium gas was used for ablation; before entering the inductively coupled plasma, argon was admixed as make-up gas. The applied laser fluence (approximately 5.5 J/cm^2^) in combination with the high stage scan speed (120 mm/s), resulting in low number of laser shots per position, was not sufficient to create negatively contributing amount of sodium signal originating from glass substrates. This was also true for other highly abundant elements in glass, such as potassium. Before measurements, a thin gold layer was deposited on every sample as a pseudo-internal standard. Samples were rasterized using a line-scan pattern that covered the complete tissue section. Laser output energy was adjusted to ablate all tissue material in one run of analysis.

### Chemometric analyses

As described in a previous paper^33^ Principal component analysis (PCA) is an unsupervised multivariate method allowing variable reduction by building linear combinations of wavenumbers varying together, called Principal Component (PC)^34^. The first principal component explains most of the data variance. The second principal component, uncorrelated to the first one, accounts for most of the residual variance and so on. Usually 2 to 6 PCs are sufficient to explain the major proportion of the original variance of the data set, reducing the description of each spectrum to 2 to 6 numbers representing the projection (scores) of each spectrum on the PCs.

Hierarchical bottom-up clustering (HCA) is a method for grouping spectra based on their similarity. It starts with the computation of a distance matrix between all spectra. The Euclidian distance was used here. The more similar (shortest distance) spectra form a cluster and the distance matrix is updated for the remaining spectra/clusters. The process is then repeated; most similar clusters are successively merged until there is only one cluster left. There is no need to define the final number of clusters. As described in Benard *et al*.^35^, K-means clustering is based on a non-hierarchical process and is particularly efficient for dealing with large data sets as it is less demanding of computational resources^36^. The number of clusters has to be defined before computation. The process minimizes the intra-cluster variance and maximizes the inter-cluster variance. The algorithm works iteratively to assign each data point to one of K groups based on the Euclidian distance. As the first step of K-means clustering starts with a random selection of centers, the final result may therefore depend on this random selection. The process was repeated 10 times to improve the robustness of the process. Two-dimensional (2D) correlation was calculated as described by Noda^37^ and used recently for the investigation of breast cancer tissue sections by FTIR imaging^29^.

Double clustering analysis is designed at providing an overview of the similarities both within spectra and between spectra. It has been intensively used for analyzing gene expression as families of genes displaying an identical behavior upon a perturbation (pathology, exposure to a drug etc.) do form functional clusters and the phenotypes (the cells for instance) are also grouped according to their gene expression. Here, both FTIR absorbance and element abundance have been scrutinized in place of gene expression. They have been sorted with a K-means clustering while spectra were sorted with a full hierarchical clustering.

All computations have been carried out with Kinetics, a custom-made program running under Matlab (Mathworks, Inc.).

### Processing of FTIR spectra

For FTIR images, processing was carried out in the following sequence: 1. water vapor contribution subtraction, 2. removal of CO_2_ contribution, 3. scaling, 4. baseline subtraction and 5. filtering for signal-to-noise ratio. The processing was reproduced for each spectrum of each image independently.

#### Subtraction of water vapor contribution

A reference water vapor spectrum was acquired as the mean of the difference between all the spectra of an image recorded in the absence of any sample before and after purging the sample cabinet with dry air. The area of the water vapor band between 1878 and 1860 cm^−1^ was used as a reference to determine the subtraction coefficient. Correction for water vapor contribution brought little visible change to the spectra as the sample cabinet was continuously purged with dry air during the experiments and as the spectra were quite intense. Nevertheless, it is critical to remove this contribution to take full advantage of the accuracy of the FTIR spectra^8^.

#### Removal of CO_2_ contribution

As CO_2_ absorbs between 2450 and 2250 cm^−1^, a region where biological molecules do not absorb, this region of the spectrum is of little interest. Correction is however required in some instances for proper scaling of the spectra on the display. Here, a straight line was drawn between 2450 and 2250 cm^−1^ to replace the CO_2_ contribution.

#### Scaling

Scaling of the spectra is necessary to account for thickness variation in the same section and among different sections. It is well documented that microtome sections have thicknesses that varies in the range of several % or even several tens of %^38^. Here, the area under the amide I and amide II bands (i.e. between 1730 and 1490 cm^−1^) has been set to an arbitrary value identical for all the spectra

#### Baseline subtraction

Baseline subtraction is required because shifts in baseline can be observed in spectra present in images. The origin of these shifts remains unclear but loss of light by reflection on top of the sample and variation in substrate reflectivity may contribute significantly to this phenomenon. The spectra were baseline-corrected. The baseline was built as a succession of segments interpolated linearly between spectral points at 3900, 3800, 3666, 3116, 3000, 2700, 1800, 1490, 1422, 1358, 1114, 1138, 980 and 900 cm^−1^ and subtracted from each spectrum. A baseline going through many points such as the one described above does not represent a “real” baseline but, applied in a consistent way, it improves the quality of spectral comparison by enhancing the significance of absorbance variations with respect to the points set to zero as demonstrated elsewhere^8^. After such a correction, it is usually not necessary to apply second derivatization as also demonstrated elsewhere^8^.

#### Signal-to-noise ratio (SNR)

Flagging spectra with insufficient Signal-to-Noise ratio (SNR) is required to eliminate spectra of poor quality from further analyses. The SNR was checked on each spectrum as described earlier^10^. Unless otherwise mentioned, it was required to be higher than 150 with noise defined as the standard deviation in the 2000–1900 cm^−1^ region of the spectrum and signal defined as the maximum of the curve between 1730 and 1490 cm^−1^ after subtracting a baseline passing through these two points.It has been discussed before^8^ that requiring high signal-to-noise ratio (SNR) is time consuming as SNR increases only as the square root of the number of scans. According to simulations made by Bhargava^39^, SNR beyond 150 provides little benefit for typical classification.

**Figure 2:**
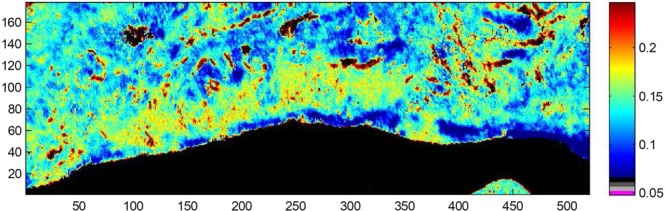
Example of a processed FTIR image. Here the ratio A^1230^/A^1655^ is reported, evidencing the epithelial cells surrounding the ducts. The pixels where the SNR is below 150 have been turned to black. This image corresponds to the framed region in the section shown in Fig. 1.

Once all the corrections have been applied (Fig. 2), one may be confident that the spectral features present in the spectra are only related to the sample.

Figure 2 reports a processed FTIR image of a region framed in Fig. 1. In this image, the absorbance at 1230 cm^−1^ representative of phosphate vibrations found in nucleic acids has been divided by the absorbance at 1655 cm^−1^ representative of proteins. Epithelial cells surrounding ducts, in red, are clearly distinguished from the rest of the tissue.

### Processing LA-ICP-MS spectra

LA-ICP-MS images have been recorded for ^13^C, ^31^P, ^34^S, ^52^Cr, ^55^Mn, ^56^Fe, ^58^Ni, ^63^Cu and ^64^Zn.

#### Background subtraction

In a first step, areas without samples were selected to obtain a background relevant to the current tissue section

**Figure 3:**
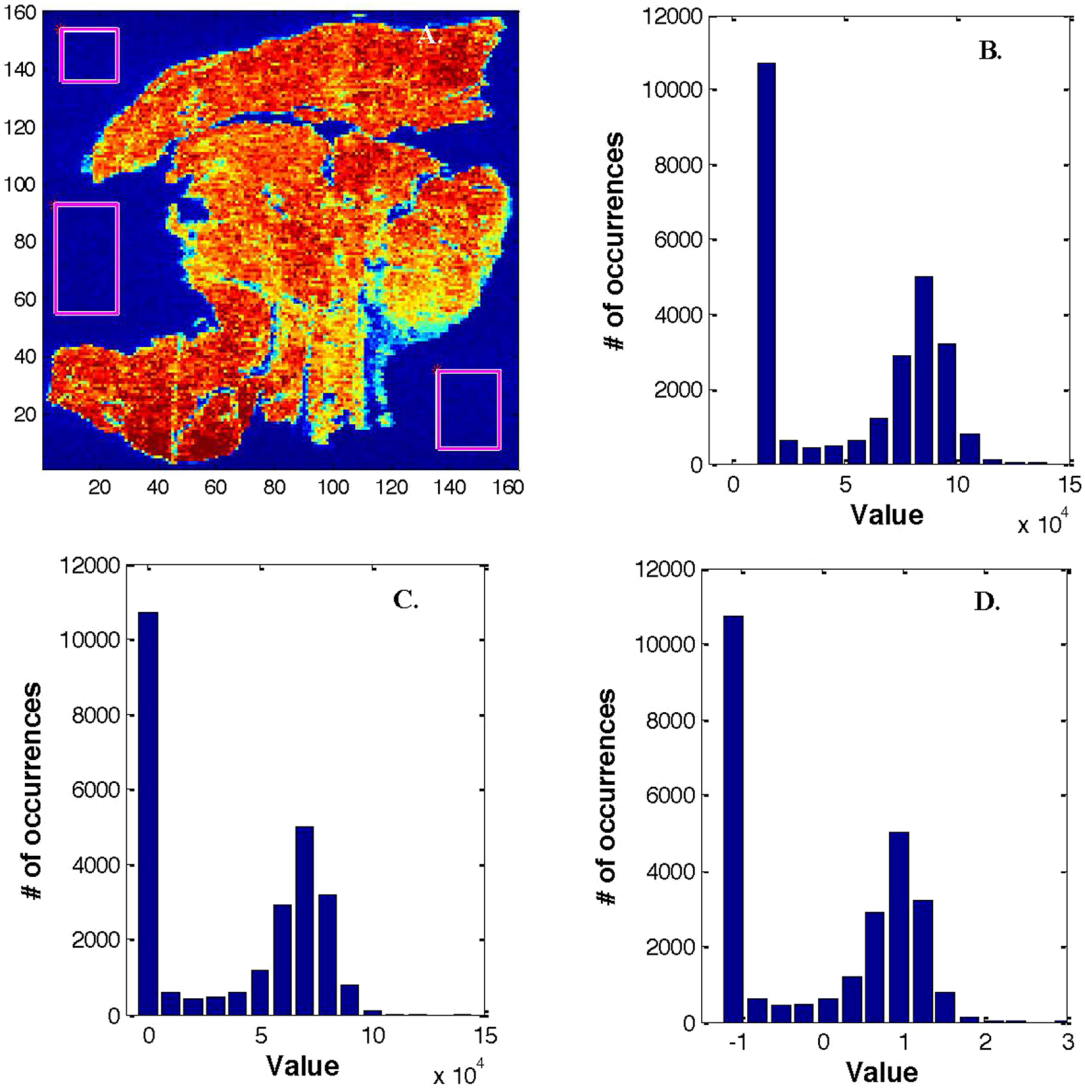
Illustration of the process followed for background subtraction. A. the rectangles represent the areas selected to be used as background in this ^13^C image. In this example, 1918 spectra were included in the rectangles and their mean was subtracted from all spectra. B. intensity distribution before subtraction of the background, C. intensity distribution after subtraction of the background, D. intensity distribution after subtraction of the mean and division by the standard deviation.

Rectangles were drawn in areas of the images where no tissue contribution was present (Fig. 3A). All spectra present in these areas were collected and averaged. The mean spectrum representing the background was then subtracted from all spectra of the image. The distribution of the intensities in the image is now shifted, bringing the large contribution of regions of the image without tissue to zero (Fig. 3B and C).

#### Scaling

On the contrary to FTIR images, the scale of the observed intensities varies widely for the different elements. Each spectrum was therefore processed by subtracting the mean and dividing by the standard deviation. For each individual image, the mean has therefore been subtracted and every spectrum of the image was divided by the standard deviation. The consequence is that the areas without tissue have usually negative values and the areas where the tissue is present have positive values as indicated by the intensity distribution (Fig. 3D).

## Results

Six breast tissue sections have been analyzed by FTIR imaging and LA-ICP-MS. These 6 tissue sections have been selected for their size which is representative of the samples analyzed in the clinic. Size is an issue, especially for FTIR imaging which collect spectra every 6.25 µm, resulting in 2.5 million full FTIR spectra per cm^2^. Most of our samples were close or above 2 cm^2^. The detail of the samples is presented in Table 1. The goal of this paper is to report in detail the combined analysis of FTIR and LA-ICP-MS images which, to the best of our knowledge, has not been attempted before. We show how images obtained by both approaches can be merged into a single data set and analyzed.

### Comparison of FTIR and LA-ICP-MS images

**Figure 4:**
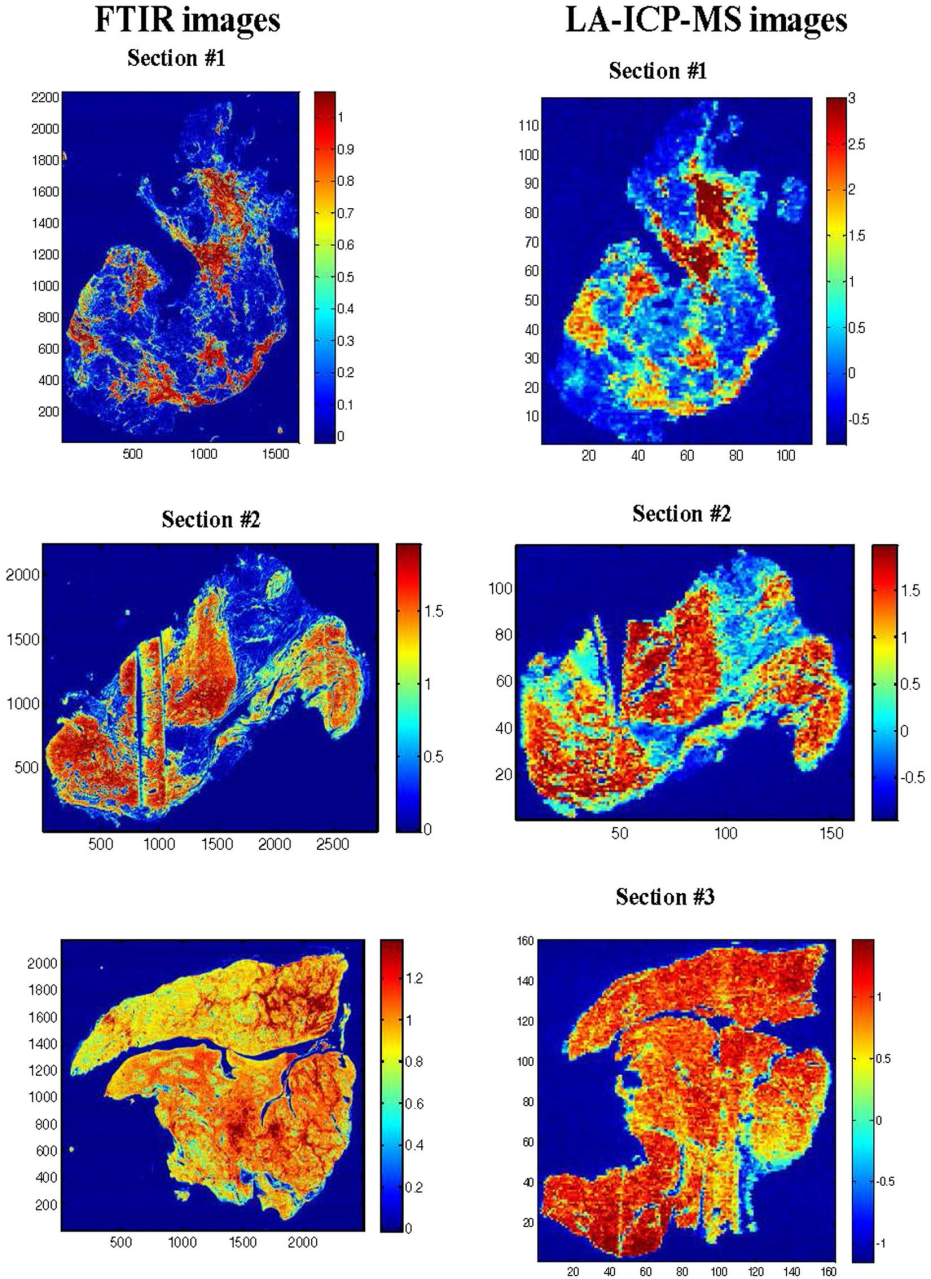
FTIR image reporting the absorbance at 1652 cm^−1^ of 3 breast tissues (left column) and elemental analysis image reporting the abundance of ^13^C for the same 3 breast tissues (right column). Data have been processed as described below in the text. Regions with SNR < 150 have been turned to dark blue.

The examples reported in Fig. 4 indicate that shape and orientation of the tissues sections are similar for FTIR and LA-ICP-MS imaging but not identical. Image registration will therefore be required for comparing identical regions between the two imaging modes^40^.

### Analysis of FTIR images

Analysis of FTIR images in the context of breast tissue has been described in numerous papers^9–12,35,41,42^ and will not be detailed here. FTIR imaging has been shown to identify successfully the various cell types present in breast tissue section^12,35,43^, to reveal breast cancer effect on the extracellular matrix^11^ and on fibroblasts^44,45^, to distinguish the different types of lymphocytes (B cells, T cells CD4+ or CD8+)^33,46,47^ and to identify most breast cancer cell lines grown *in vitro* after FFPE processing^48^ or in spheroids^13^. It has also been shown to be able to classify anticancer drug effects according to the drug-induced spectral perturbations observed on cancer cell lines^49^. In the framework of this study, the FTIR images will only be used in conjunction with LA-ICP-MS images.

### Analysis of LA-ICP-MS images

#### Resizing and stitching LA-ICP-MS images

**Figure 5:**
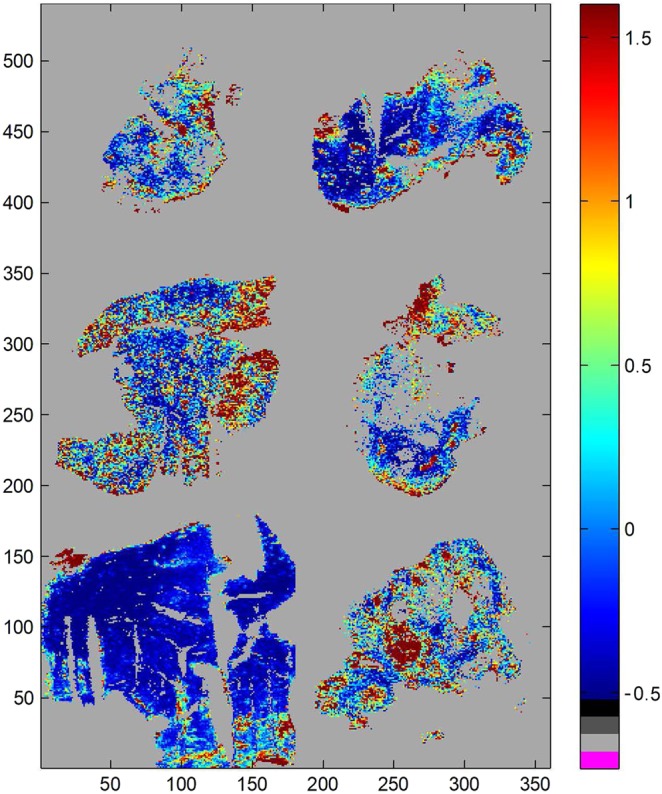
^64^Zn distribution in the 6 tissue sections described in Table 1. The areas in grey have values below 0 for both ^13^C and ^64^Zn.

The principal interest of imaging of tissue is to compare element abundance not only within a tissue section but also among various tissue sections. To allow such a comparison, the individual LA-ICP-MS images have been padded with zeros on the left and right as well as below and above the actual image to obtain a final image size of 180 × 180 pixels for all tissue sections. Only section #5 (see Table 1) had to be cut on the edges to fit into this common size. The resized images were then assembled into a unique matrix containing the 6 tissue section images (Fig. 5).

Once the individual images have been merged into a larger single image matrix, comparison can be carried out. A normalisation by the standard deviation for each element was applied on the new larger image for proper comparison between tissue sections. Figure 5 reports ^64^Zn distribution. It must be stressed that the analysis of the spectra (we also use here the term “spectrum” for the abundance profile of the elements) now required a filter allowing to separate spectra belonging to tissues and spectra belonging to regions outside the tissue sections. Here each spectrum with a value below 0 has been assigned to non-tissue response and appears in grey. It can be observed (not shown) that the same filtering is obtained when using ^13^C values. Figure 5 clearly indicates that it is a reasonable filter to apply.

It is interesting to note that distribution of some elements such as ^64^Zn reported in Fig. 5 is not homogeneous. The distribution maps for ^13^C, ^31^P, ^34^S, ^52^Cr, ^55^Mn, ^56^Fe, ^58^Ni, ^63^Cu and ^64^Zn can be found in Fig. S1.

Correlations between the abundance of the different elements can be addressed in two ways: correlation analysis and principal component analysis.

#### Correlation analysis

**Figure 6:**
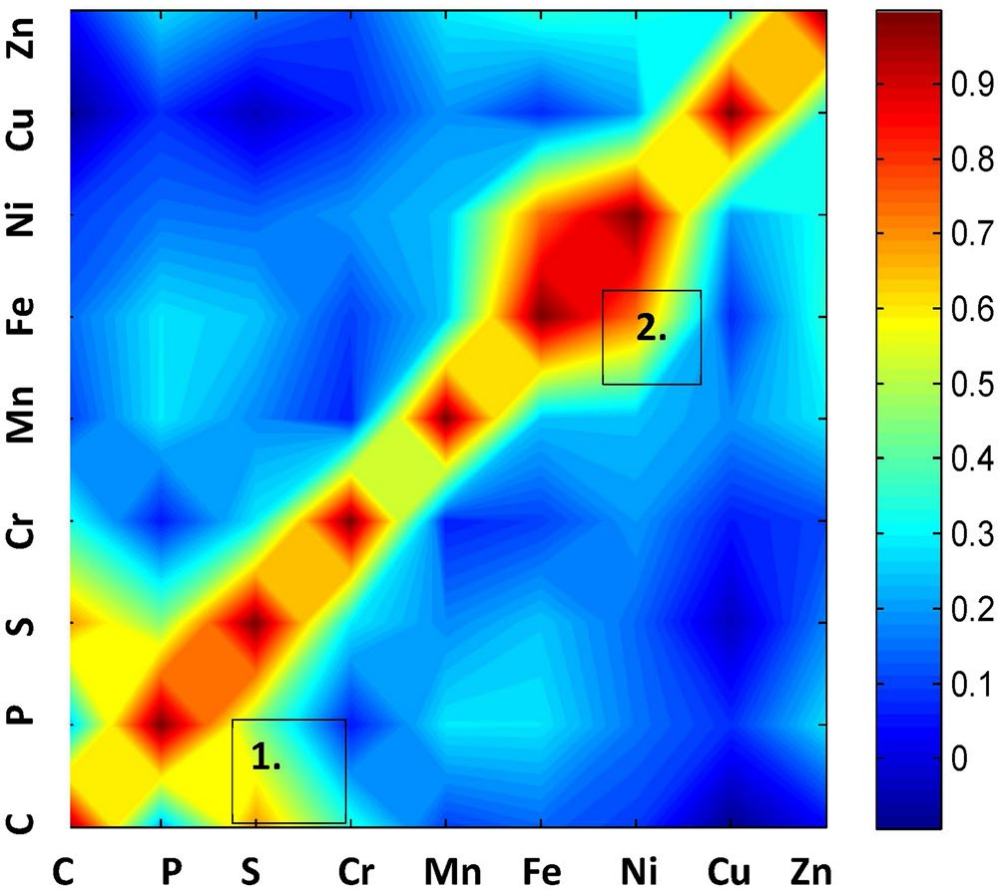
**:** 2D correlation analysis of the abundance of elements (^13^C, ^31^P, ^34^S, ^52^Cr, ^55^Mn, ^56^Fe, ^58^Ni, ^63^Cu and ^64^Zn) in the 6 breast tissue sections.

It is first important to select only spectra and element values which belong to tissue. For this purpose, only spectra with positive values for ^13^C and ^64^Zn (see Fig. 5) were retained. All the 57,892 spectra on a total of 194,400 were selected. For correlation analysis, the correlation coefficient was computed between all elements. The result is reported in Fig. 6.

The diagonal indicates that, as expected, each element is correlated with itself. Off-diagonal cross peaks indicate the presence of two strong correlations 1) between ^13^C and ^34^S (label 1 in Fig. 6) and 2) between ^56^Fe and ^58^Ni (label 2).

#### Principal component analysis

**Figure 7:**
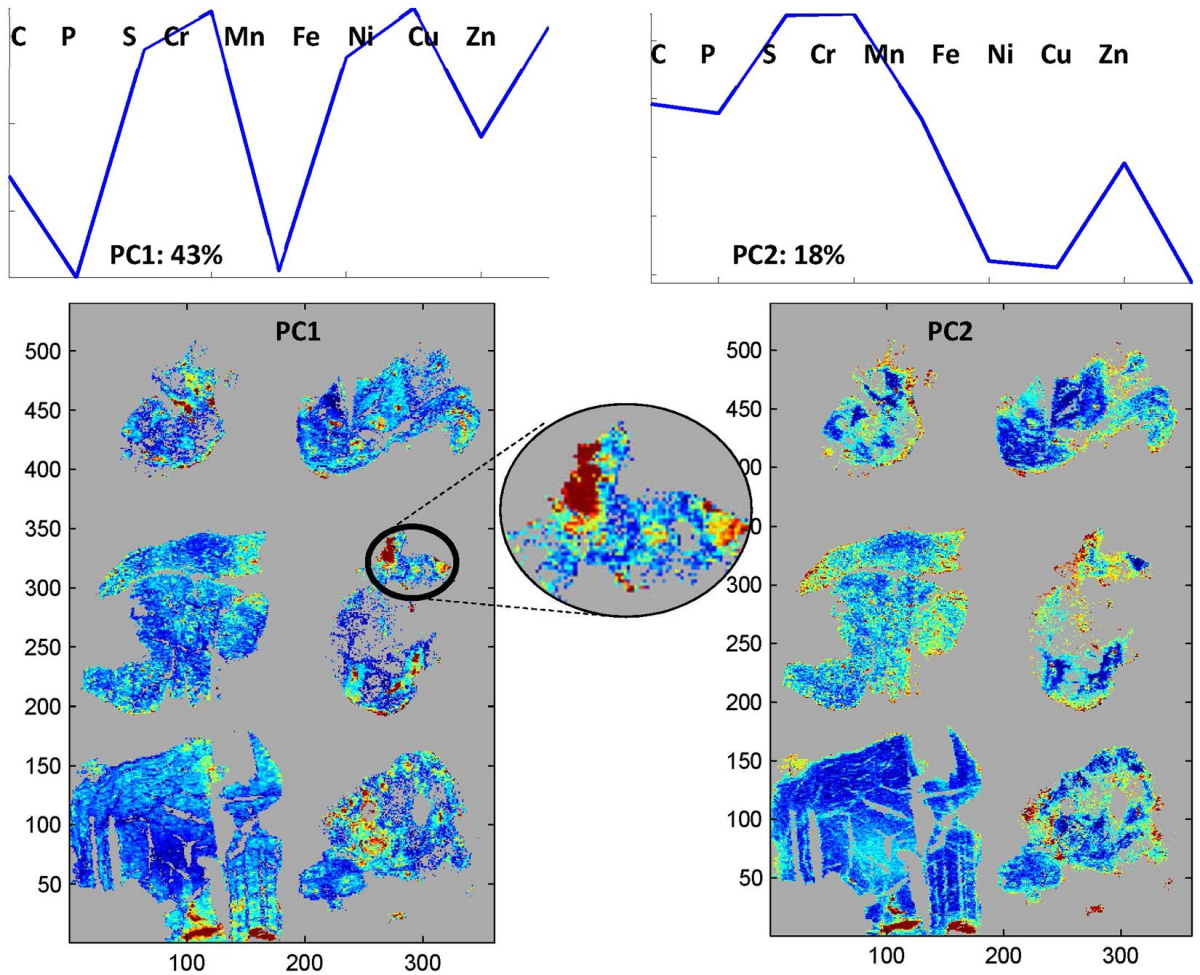
Top: shape of the first 2 principal components PC1 and PC2. Bottom score maps for PC1 and PC2 of 6 tissue sections. PCA was computed only on the spectra with ^13^C values above 0 as shown in Fig. 5.

Principal component analysis was performed on the spectra of the 6 breast tissue sections analysed above. Figure 7 reports score maps for the first 2 principal components as well as the shape of these 2 principal components. PCA was performed only on element distributions belonging to the tissue.

It is interesting to analyse the shape of the first PCs. PC1 describes a correlation between ^34^S and ^52^Cr as well as between ^56^Fe, ^58^Ni and ^64^Zn while ^31^P and ^55^Mn abundance varied in the opposite direction. The enlargement in Fig. 7 demonstrates that PC1 identifies regions of the images where large concerted variations of these elements do occur. PC1 describes the largest part of the variance, i.e. 43% of the total variance, and is orthogonal to all other sources of variance described by the other PCs. PC2 describes a correlation between ^34^S and ^52^Cr varying in the opposite direction as compared to ^56^Fe, ^58^Ni and ^64^Zn. It represents 18% of the total variance. All other PCs account for 10% or less of the total variance. It is interesting to note that the details revealed by PCA were not apparent in the previous global correlation analysis which considers only the overall correlations.

### Co-analysis of LA and FTIR data

As mentioned above, FTIR and LA-ICP-MS are orthogonal methods providing information on respectively organic molecules and inorganic elements. Their co-analysis could therefore reveal a relevant discrimination power higher than for each method considered alone. The problems related to co-analysis and the solution developed to solve them will be illustrated with one tissue section (section #3 in Table 1).

#### Image processing

In the first step, a matching sub-region of the LA-ICP-MS and FTIR images was extracted for both image types. Yet, overlay of the image required both a rotation of one image with respect to the other and a pixel resolution match. It was decide to modify the FTIR images whose pixel resolution was much higher. Rotation was obtained by applying a rotation matrix ([cosθ −sinθ; sinθ cosθ]) on the pixel coordinates and interpolating the values accordingly. A rotation by 2° was applied. Resampling was obtained first by binning pixels to arrive at a pixel number along X and Y axes slightly above the one of the LA-ICP-MS image. In a second step, 2D-Fourier transform of the image was computed for the images representing spectral intensities wavenumber by wavenumber. At each wavenumber, the image FT was cut for keeping the final number of points and a FT^−1^ was taken to generate the absorbance image with the right pixel resolution. The process was repeated for each wavenumber, thereby recreating a series of spectra. As a result, the two images can now be superimposed and have the same number of pixels in X and Y directions. In order to merge the two approaches, the next step was to fuse the data of the two images into a single matrix.

#### Concatenation of FTIR and LA image data

**Figure 8:**
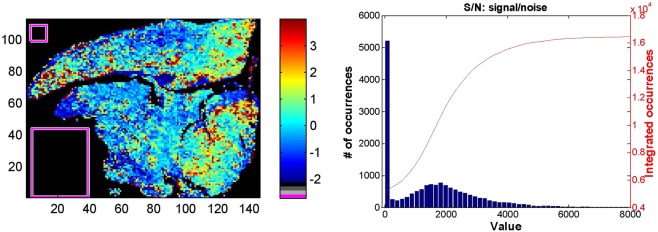
Left: represents the ^64^Zn/A^1654^ FTIR ratio. The two rectangles include a total of 1705 spectra whose average was subtracted from all spectra of the image. For all values at each wavenumber/element, the mean was subtracted and it was divided by the standard deviation. Right: distribution of the SNR through the FTIR image. The red curve reports the integrated counts.

To obtain a single matrix of data, the two matrices (FTIR and LA-ICP-MS) were concatenated. The spectra now consist for one part in infrared absorbance and, for the other, in a measure of the 9 element abundance. As the units are unrelated for FTIR and LA-ICP-MS, a normalisation by the standard deviation was applied for the new data set. First a background specific to this section was subtracted by subtracting the mean of the spectra present in an area without tissue (Fig. 8), then for each wavenumber and each element, the mean value was subtracted and the resulting value was divided by the standard deviation. The process is illustrated in Fig. 8 which presents the ratio between ^64^Zn abundance and protein quantity as measured by the absorbance at 1654 cm^−1^.

It must be stressed that the averaging of the FTIR applied to create larger pixels resulted in a data set with an excellent signal-to-noise ratio (SNR) centred around 1800 (Fig. 8).

#### Correlation analysis

**Figure 9:**
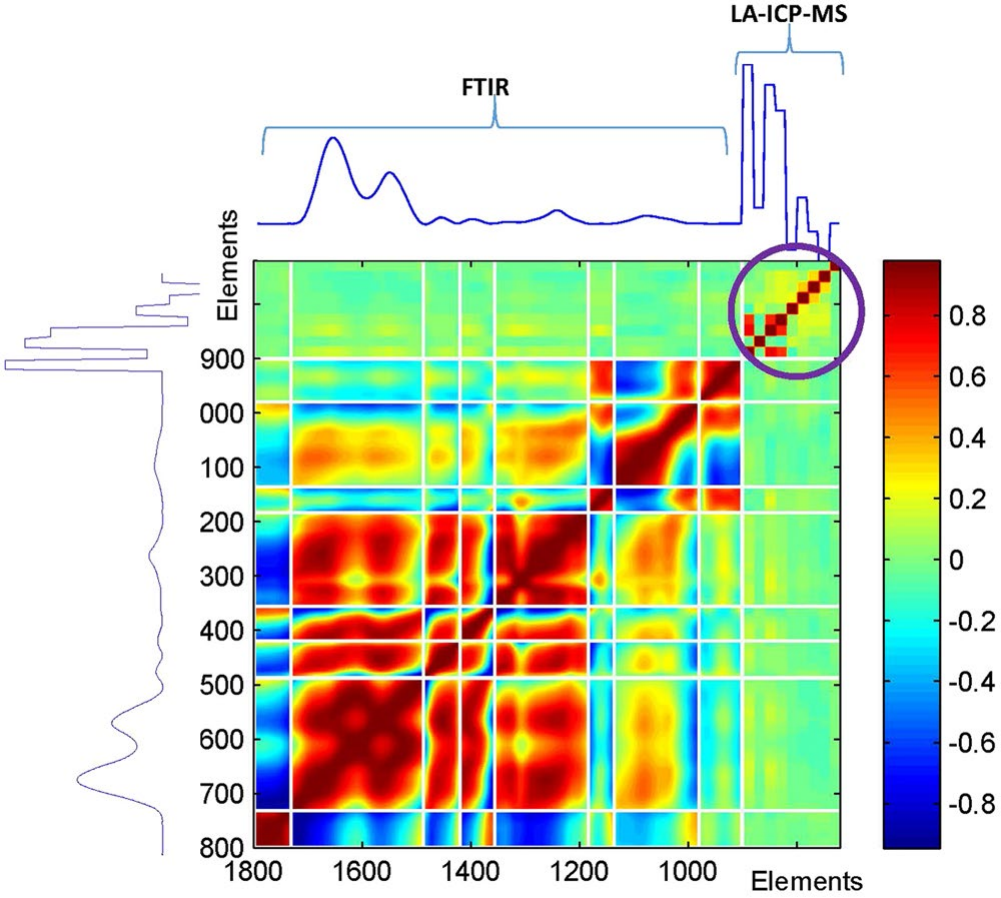
Correlation analysis of the FTIR/LA-ICP-MS concatenate spectra. The LA-ICP-MS data are represented by 9 points present below 900 cm^−1^ as indicated by the purple circle. Elements are in the same sequence as previously: ^13^C, ^31^P, ^34^S, ^52^Cr, ^55^Mn, ^56^Fe, ^58^Ni, ^63^Cu and ^64^Zn. The white line present on the figure corresponds to points of the spectra where there is no variance because a baseline has been drawn.

As LA-ICP-MS data contain only 9 points (9 elements) while FTIR data contain 226 points between 1800 and 900 cm^−1^ after interpolating the FTIR spectra to obtain one data point every 4 cm^−1^, each LA-ICP-MS data point has been quintupled. It makes correlation analysis more clearly readable and gives a significant weight to LA-ICP-MS data in PCA. Figure 9 reports the correlation map.

Observation of Fig. 9 indicates that there are significant correlations within the FTIR spectra, particularly well-marked after normalization by the standard deviation but little correlation between LA-ICP-MS and FTIR bands. It is very interesting that little significant correlation exists between FTIR and LA-ICP-MS data, demonstrating the very good complementarity between the two approaches.

#### PCA

**Figure 10:**
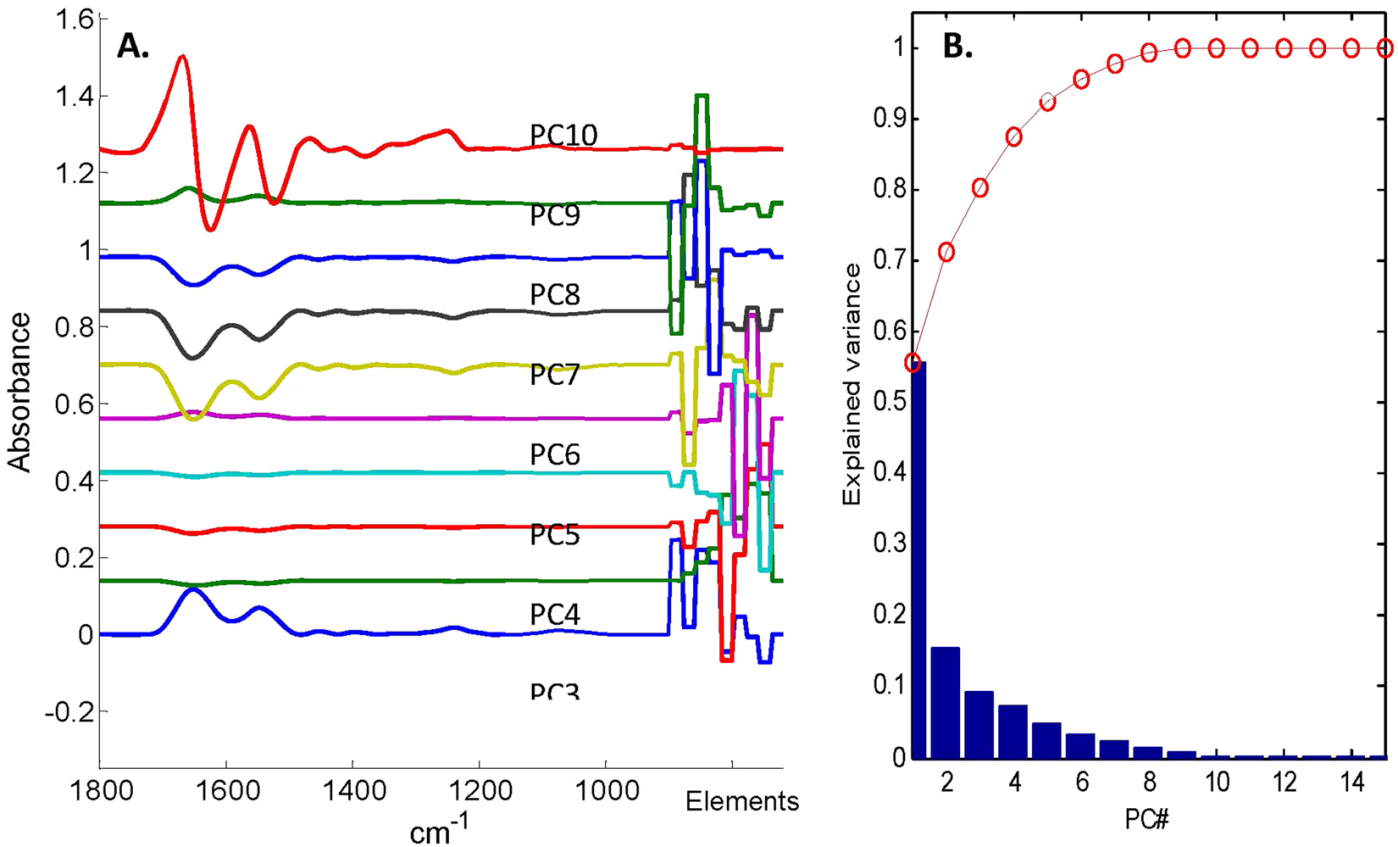
(**A**) PCs 1 to 10 (from bottom to top) obtained after PCA of the data presented in Fig. 8. The mean spectrum has not been subtracted prior to PCA. (**B**) fraction of the variance explained as a function of the number of PCs. The red line reports the cumulative fraction of the variance explained.

Principal component analysis (Fig. 10) also indicates that within this particular image, there is little correlation between element distribution and FTIR bands. As here the mean spectrum has not been subtracted before PCA, the first PC (bottom, blue) represents the mean of the data. The next 4 PCs describe essentially uncorrelated abundance variations of various elements with no significant correlation with FTIR features. PCs 6, 7 and 8 on the other hand describe correlated variations in LA-ICP-MS and FTIR spectral features but describe only less than 5% of the total variance (Fig. 10B). The last PC shown shows variations in the FTIR spectrum not significantly correlated with element variations.

#### Double clustering analysis

**Figure 11:**
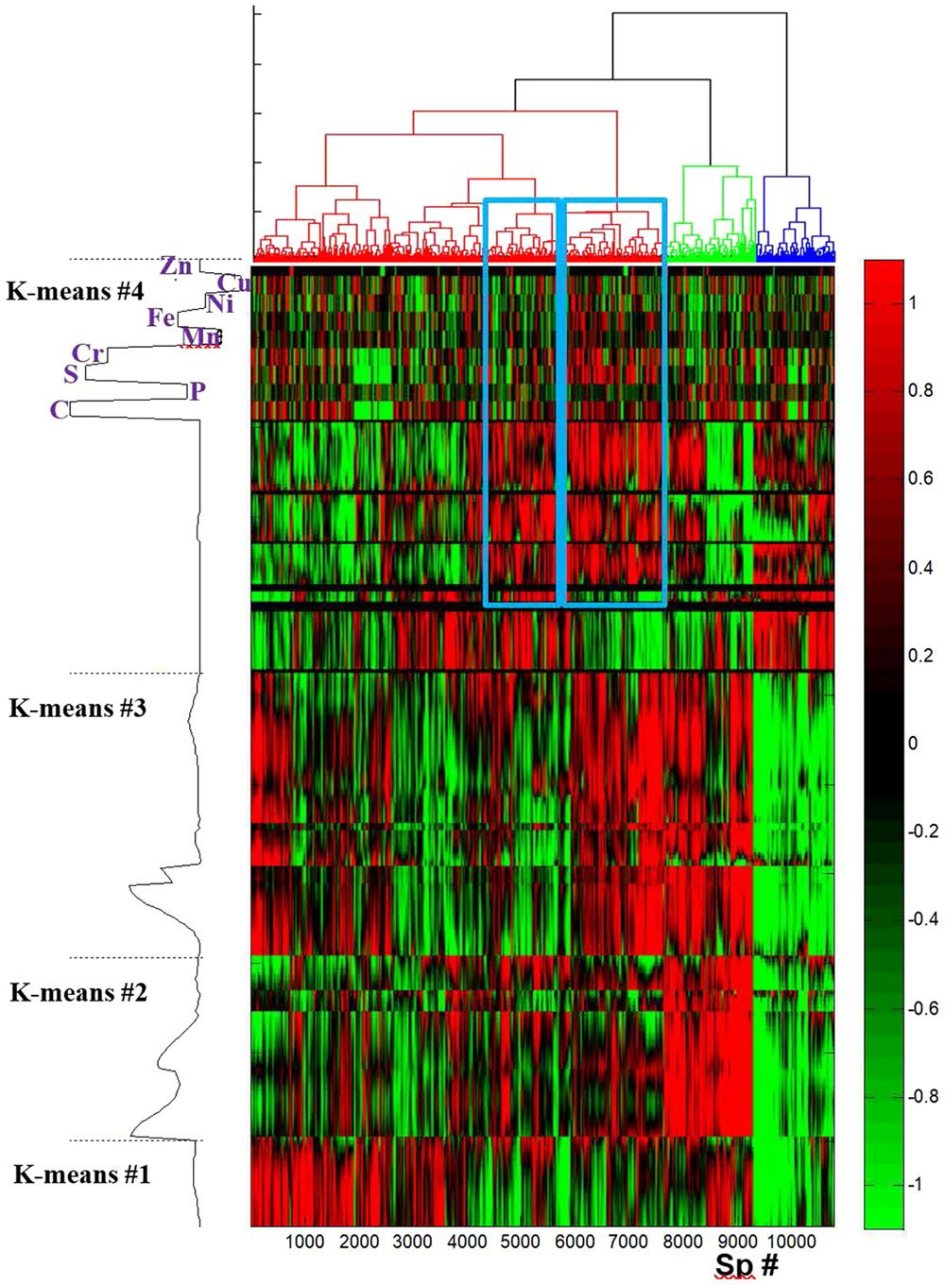
Representation of the intensities of the 10,780 FTIR/LA-ICP-MS spectra of section #3 presented on Fig. 8 passing a SNR threshold of 500 after double clustering analysis. Spectra were processed by subtraction of the mean and normalization by the standard deviation prior to clustering. The 10,780 spectra were sorted according to a hierarchical cluster analysis shown on top of the figure. The wavenumbers/elements were sorted in 4 clusters by K-means clustering. The dotted line on the left side of the figure indicates the limits of the clusters. The mean spectrum after sorting the wavenumbers/elements by the K-means (“sorted spectrum”) is also presented on the left side of the figure. For the sake of the clarity, the “sorted spectrum” is shown prior to mean subtraction and normalization by standard deviation.

Double clustering analysis is commonly used when analysing gene transcription data. First, the mean spectrum has been subtracted from all spectra (merged FTIR/LA-ICP-MS data sets, see Fig. 8) and each value was normalized by the standard deviation. In a second step, the so-processed merged FTIR / LA-ICP-MS spectra have first been sorted according to a hierarchical cluster analysis. The spectral features (wavenumbers and elements) have then been sorted according to a K-means cluster analysis. Figure 11 reports the intensity of the sorted values.

Wavenumbers/elements clustering was obtained by the K-means method after mean subtraction and normalization by standard deviation. The limits of the clusters and the mean spectrum obtained after sorting the wavenumbers/elements appear on the left hand side of the figure. For the sake of the clarity, the sorted spectrum is shown (on the left hand side of the figure) prior to mean subtraction and normalization by standard deviation. K-means #1 cluster contains the FTIR spectral region 1070–1020 cm^−1^, in K-means #2 cluster, the right hand side of Amide I (wavenumbers <1645 cm^−1^) and the full Amide II bands can be recognized as well as 1380–1430 cm^−1^ region, K-means #3 cluster contains the left hand side of Amide I (wavenumbers >1645 cm^−1^) and 1380–1180 cm^−1^ region. K-means #4 cluster contain the FTIR spectral region found between 1020 and 900 cm^−1^ as well as all elements. Figure 11 reveals some correlations that were not apparent when looking at the entire dataset. An example is indicated by the two spectrum clusters identified by the blue rectangles on Fig. 11. In these particular clusters of spectra, wavenumber/element cluster #4 groups high values for ^13^C, ^34^S and ^52^Cr and the 1020–900 cm^−1^ FTIR spectral region assigned to glycosylation and phosphate vibrations.

## Discussion

For the analysis of tissue sections, some features of infrared imaging are particularly interesting. One of these advantages is that it is fully FFPE (formalin-fixed, paraffin-embedded) compatible. Currently, FFPE remains the standard for clinical histopathology. Samples are stable and the large library of FFPE tissues allows retrospective studies. Yet, while the morphology of the tissues is well preserved upon formalin fixation and paraffin embedding, nucleic acids are usually partially deteriorated, making NGS (new generation sequencing) and transcriptomic studies difficult. LA-ICP-MS can also be applied to tissue sections and provide new information on the tissue. So far it has been essentially used to help immunochemistry imaging^50^ or to locate platinum-based anticancer drugs in tissues^51^ but relatively few works deal with measuring biologically relevant elements in tissue sections^52^. We previously showed on breast tumor that FTIR spectroscopy has a high potential to identify tissue types^10,35^ but we also showed that many FTIR biomarkers are highly correlated^29^. We also considered both FTIR and LA-ICP-MS for investigation of rat brain after ischemic stroke but the data were collected and analysed separately^53^. While FTIR imaging has a demonstrated use for diagnostics and prognostics in breast cancer, LA-ICP-MS is a completely orthogonal method that could complement FTIR with another set of markers. A key result obtained in this paper is the correlation analysis (Fig. 9) which indicates that there is no significant correlation between FTIR data and elemental analysis. Quite significant correlations exists within the FTIR data set as indicated on Fig. 8. Similarly, some correlation exists between the abundance of different elements (Fig. 6). Yet, almost no correlation is found between the two techniques (Fig. 9). This is confirmed by the PCA analysis reported in Fig. 10 which displays little covariance between the two methods before PC#6 (PC#1 is the mean). The LA-ICP-MS method brings therefore new non-redundant data which can only help potential diagnostics. Even though it was not the purpose of the present paper to decipher a diagnostic tool, a useful contribution of elemental analysis to diagnostic is supported by the role trace element have in some enzymes involved in disease progression, e.g. metalloproteinases^54^ as well as in many zinc finger motives involved in reprogramming breast cancer transcriptional network^55,56^ related to metastasis.

For microscopy approaches, resolution is an issue. As reviewed elsewhere^8^ for FTIR imaging, resolution is diffraction-limited, which means intracellular details will generally not be resolved^57,58^. Furthermore, pixel content may also be affected by the point spread function of the Schwarzschild optics^58,59^. The optimal size of the pixels has been evaluated by Reddy *et al*.^60^. Roughly, the wavelength (5–10 µm for the spectral range considered in this study) places a limit to the expected spatial resolution. Though there are means to record infrared images at much higher resolution, they are not practically usable when several cm^2^ have to be analyzed. Yet, numerous studies quoted before in this paper have demonstrated the usefulness of FTIR imaging for the analysis of tissue sections. When looking at essential trace elements, a resolution of 50 μm is a reasonable compromise between resolution and sensitivity^61,62^. Though single cell analysis is out of reach, pathologies like cancer usually display sufficient cell density to allow a precise characterization of the cell type. The LA-ICP-MS technique can therefore give sufficient sensitivity and spatial resolution to link the elemental data with the molecular data obtained from the FTIR imaging in cancer pathologies. Similarly, characterization of changes in the extracellular matrix, already shown to be feasible by FTIR imaging^11,41,45^, is perfectly adapted to characterization by LA-ICP-MS.

It must be stressed here that the goal of the paper was to describe how FTIR and LA-ICP-MS imaging data can be combined and analyzed simultaneously to provide a larger set of markers. We used a set of 6 breast cancer tissues with different pathologies (Table 1). The samples were selected for their within-image and between-image diversity of tissues. Within this sampling, we could conclude the elemental markers do not significantly covariate with the FTIR markers, underlining the complementarity between the two methods.

In conclusion, the results obtained in this paper show the feasibility of merging FTIR and LA-ICP-MS datasets, providing a hybrid set of markers based respectively on organic molecules and on trace elements. The correlation analyses and PCA presented in the paper show that little correlation could be found here between FTIR and LA-ICP-MS values. In the limited size of the sampling tested, this is a good indication that both do not co-vary and therefore bring their own independent information. Interestingly, in a recent paper, Anyz *et al*.^31^ developed a similar concept to compare adequately LA-ICP-MS images and H&E-stained section images. Their goal was to better relate abundance of Cu and Zn to histological features. The present paper add the FTIR dimension which contains a demonstrated series of biomarkers. The next step will be to repeat the analysis on a much large selection of tissues more specific pathologies.

## Supplementary information

S1

## Data Availability

The datasets used and/or analysed during the current study are available from the corresponding author on reasonable request.

## References

[1] Al-Othman S (2015). Tackling cancer control in the Gulf Cooperation Council Countries. Lancet Oncol..

[2] Prat A, Perou CM (2011). Deconstructing the molecular portraits of breast cancer. Mol. Oncol..

[3] Cleary AS, Leonard TL, Gestl SA, Gunther EJ (2014). Tumour cell heterogeneity maintained by cooperating subclones in Wnt-driven mammary cancers. Nature.

[4] Yates LR (2015). Subclonal diversification of primary breast cancer revealed by multiregion sequencing. Nat. Med..

[5] Mao Y, Keller ET, Garfield DH, Shen K, Wang J (2013). Stromal cells in tumor microenvironment and breast cancer. Cancer Metastasis Rev..

[6] Chichon AA, Degnim AC, Visscher DW, Radisky DC (2010). Microenvironmental Influences that Drive Progression from Benign Breast Disease to Invasive Breast Cancer. Journal of Mammary Gland Biology and Neoplasia.

[7] Baker MJ (2014). Using Fourier transform IR spectroscopy to analyze biological materials. Nat. Protoc..

[8] Goormaghtigh E (2016). Infrared imaging in histopathology: is a unified approach possible?. Biomed. Spectrosc. Imaging.

[9] Holton SE, Bergamaschi A, Katzenellenbogen BS, Bhargava R (2014). Integration of molecular profiling and chemical imaging to elucidate fibroblast-microenvironment impact on cancer cell phenotype and endocrine resistance in breast cancer. PLoS One.

[10] Verdonck M (2016). Characterization of human breast cancer tissues by infrared imaging. Analyst.

[11] Kumar S, Desmedt C, Larsimont D, Sotiriou C, Goormaghtigh E (2013). Change in the microenvironment of breast cancer studied by FTIR imaging. Analyst.

[12] Mittal Shachi, Yeh Kevin, Leslie L. Suzanne, Kenkel Seth, Kajdacsy-Balla Andre, Bhargava Rohit (2018). Simultaneous cancer and tumor microenvironment subtyping using confocal infrared microscopy for all-digital molecular histopathology. Proceedings of the National Academy of Sciences.

[13] Smolina M, Goormaghtigh E (2018). Gene expression data and FTIR spectra provide a similar phenotypic description of breast cancer cell lines in 2D and 3D cultures. Analyst.

[14] Bird B (2009). Detection of breast micro-metastases in axillary lymph nodes by infrared micro-spectral imaging. Analyst.

[15] Fabian H, Lasch P, Naumann D (2005). Analysis of biofluids in aqueous environment based on mid-infrared spectroscopy. J. Biomed. Opt..

[16] Walsh Michael J., Holton Sarah E., Kajdacsy-Balla Andre, Bhargava Rohit (2012). Attenuated total reflectance Fourier-transform infrared spectroscopic imaging for breast histopathology. Vibrational Spectroscopy.

[17] Ooi GJ (2008). Fourier transform infrared imaging and small angle x-ray scattering as a combined biomolecular approach to diagnosis of breast cancer. Med. Phys..

[18] Kallenbach-Thieltges A (2013). Immunohistochemistry, histopathology and infrared spectral histopathology of colon cancer tissue sections. J. Biophotonics.

[19] Lasch P, Haensch W, Naumann D, Diem M (2004). Imaging of colorectal adenocarcinoma using FT-IR microspectroscopy and cluster analysis. Biochim. Biophys. Acta.

[20] Nallala J (2013). Infrared imaging as a cancer diagnostic tool: introducing a new concept of spectral barcodes for identifying molecular changes in colon tumors. Cytometry. A.

[21] Bird B (2012). Infrared spectral histopathology (SHP): a novel diagnostic tool for the accurate classification of lung cancer. Lab. Invest..

[22] German MJ (2006). Infrared spectroscopy with multivariate analysis potentially facilitates the segregation of different types of prostate cell. Biophys. J..

[23] Baker MJ (2008). FTIR-based spectroscopic analysis in the identification of clinically aggressive prostate cancer. Br. J. Cancer.

[24] Baker MJ (2009). Investigating FTIR based histopathology for the diagnosis of prostate cancer. J. Biophotonics.

[25] Gazi Ehsan, Baker Matthew, Dwyer John, Lockyer Nicholas P., Gardner Peter, Shanks Jonathan H., Reeve Roy S., Hart Claire A., Clarke Noel W., Brown Michael D. (2006). A Correlation of FTIR Spectra Derived from Prostate Cancer Biopsies with Gleason Grade and Tumour Stage. European Urology.

[26] Walsh MJ (2008). FTIR Microspectroscopy Coupled with Two-Class Discrimination Segregates Markers Responsible for Inter- and Intra-Category Variance in Exfoliative Cervical Cytology. Biomark. Insights.

[27] Wood BR, Bambery KR, Evans CJ, Quinn MA, Mcnaughton D (2006). A three-dimensional multivariate image processing technique for the analysis of FTIR spectroscopic images of multiple tissue sections. BMC Med. Imaging.

[28] Walsh MJ (2007). IR microspectroscopy: potential applications in cervical cancer screening. Cancer Lett..

[29] Ali Mohamed H., Rakib Fazle, Al-Saad Khalid, Al-Saady Rafif, Lyng Fiona M., Goormaghtigh Erik (2018). A simple model for cell type recognition using 2D-correlation analysis of FTIR images from breast cancer tissue. Journal of Molecular Structure.

[30] de Vega RG (2018). Multimodal laser ablation/desorption imaging analysis of Zn and MMP-11 in breast tissues. Anal. Bioanal. Chem..

[31] Anyz J (2017). Spatial mapping of metals in tissue-sections using combination of mass-spectrometry and histology through image registration. Sci. Rep..

[32] Balbekova Anna, Lohninger Hans, van Tilborg Geralda A.F., Dijkhuizen Rick M., Bonta Maximilian, Limbeck Andreas, Lendl Bernhard, Al-Saad Khalid A., Ali Mohamed, Celikic Minja, Ofner Johannes (2017). Fourier Transform Infrared (FT-IR) and Laser Ablation Inductively Coupled Plasma–Mass Spectrometry (LA-ICP-MS) Imaging of Cerebral Ischemia: Combined Analysis of Rat Brain Thin Cuts Toward Improved Tissue Classification. Applied Spectroscopy.

[33] Wald N., Bordry N., Foukas P.G., Speiser D.E., Goormaghtigh E. (2016). Identification of melanoma cells and lymphocyte subpopulations in lymph node metastases by FTIR imaging histopathology. Biochimica et Biophysica Acta (BBA) - Molecular Basis of Disease.

[34] Johnson, R. A. & Wichern, D. W. Clustering methods and ordination. in Applied Multivariate Statistical Analysis 726–799 (Prentice Hall, 1998).

[35] Benard Audrey, Desmedt Christine, Smolina Margarita, Szternfeld Philippe, Verdonck Magali, Rouas Ghizlane, Kheddoumi Naima, Rothé Françoise, Larsimont Denis, Sotiriou Christos, Goormaghtigh Erik (2014). Infrared imaging in breast cancer: automated tissue component recognition and spectral characterization of breast cancer cells as well as the tumor microenvironment. The Analyst.

[36] Wang L, Mizaikoff B (2008). Application of multivariate data-analysis techniques to biomedical diagnostics based on mid-infrared spectroscopy. Anal. Bioanal. Chem..

[37] Noda I, Dowrey AE, Marcott C, Story GM, Ozaki Y (2000). Generalized two-dimensional correlation spectroscopy. Appl. Spectrosc..

[38] Zimmermann M (2009). Improved reproducibility in preparing precision-cut liver tissue slices. Cytotechnology.

[39] Bhargava R (2007). Towards a practical Fourier transform infrared chemical imaging protocol for cancer histopathology. Anal. Bioanal. Chem..

[40] Yang C (2015). Fully automated registration of vibrational microspectroscopic images in histologically stained tissue sections. BMC Bioinformatics.

[41] Smolina M, Goormaghtigh E (2016). FTIR imaging of the 3D extracellular matrix used to grow colonies of breast cancer cell lines. Analyst.

[42] Pounder F. Nell, Reddy Rohith K., Bhargava Rohit (2016). Development of a practical spatial-spectral analysis protocol for breast histopathology using Fourier transform infrared spectroscopic imaging. Faraday Discussions.

[43] Fernandez DC, Bhargava R, Hewitt SM, Levin IW (2005). Infrared spectroscopic imaging for histopathologic recognition. Nat. Biotechnol..

[44] Kumar Saroj, Shabi Thankaraj Salammal, Goormaghtigh Erik (2014). A FTIR Imaging Characterization of Fibroblasts Stimulated by Various Breast Cancer Cell Lines. PLoS ONE.

[45] Holton SE, Walsh MJ, Kajdacsy-Balla A, Bhargava R (2011). Label-free characterization of cancer-activated fibroblasts using infrared spectroscopic imaging. Biophys. J..

[46] Verdonck M, Garaud S, Duvillier H, Willard-Gallo K, Goormaghtigh E (2015). Label-free phenotyping of peripheral blood lymphocytes by infrared imaging. Analyst.

[47] Wald N, Legat A, Meyer C, Speiser DE, Goormaghtigh E (2015). An infrared spectral signature of human lymphocyte subpopulations from peripheral blood. Analyst.

[48] Verdonck M (2013). Breast cancer and melanoma cell line identification by FTIR imaging after formalin-fixation and paraffin-embedding. The Analyst.

[49] Mignolet A, Derenne A, Smolina M, Wood BR, Goormaghtigh E (2016). FTIR spectral signature of anticancer drugs. Can drug mode of action be identified?. Biochim. Biophys. Acta - Proteins Proteomics.

[50] Seuma J (2008). Combination of immunohistochemistry and laser ablation ICP mass spectrometry for imaging of cancer biomarkers. Proteomics.

[51] Moraleja I (2018). An approach for quantification of platinum distribution in tissues by LA-ICP-MS imaging using isotope dilution analysis. Talanta.

[52] Feng L, Wang J, Li H, Luo X, Li J (2017). A novel absolute quantitative imaging strategy of iron, copper and zinc in brain tissues by Isotope Dilution Laser Ablation ICP-MS. Anal. Chim. Acta.

[53] Ali, M. H. M. *et al*. Fourier-Transform Infrared Imaging Spectroscopy and Laser Ablation -ICPMS New Vistas for Biochemical Analyses of Ischemic Stroke in Rat Brain. *Front*. *Neurosci*. **12** (2018). 10.3389/fnins.2018.00647 PMC6157330 30283295

[54] Holanda AOdoN (2017). Zinc and metalloproteinases 2 and 9: What is their relation with breast cancer?. Rev. Assoc. Med. Bras..

[55] Zeng YF, Sang J (2017). Five zinc finger protein 350 single nucleotide polymorphisms and the risks of breast cancer: a meta-analysis. Oncotarget.

[56] Takaku M (2018). GATA3 zinc finger 2 mutations reprogram the breast cancer transcriptional network. Nat. Commun..

[57] Bhargava R (2012). Infrared spectroscopic imaging: the next generation. Appl. Spectrosc..

[58] Lasch P, Naumann D (2006). Spatial resolution in infrared microspectroscopic imaging of tissues. Biochim. Biophys. Acta.

[59] Mattson EC, Nasse MJ, Rak M, Gough KM, Hirschmugl CJ (2012). Restoration and spectral recovery of mid-infrared chemical images. Anal. Chem..

[60] Reddy RK, Walsh MJ, Schulmerich MV, Carney PS, Bhargava R (2013). High-definition infrared spectroscopic imaging. Appl. Spectrosc..

[61] Kindness A, Sekaran CN, Feldmann J (2003). Two-dimensional mapping of copper and zinc in liver sections by laser ablation-inductively coupled plasma mass spectrometry. Clin. Chem..

[62] Corbin BD (2008). Metal Chelation and Inhibition of Bacterial Growth in Tissue Abscesses. Science (80-.)..

