# Supplementary material for: An Innovative Platform Merging Elemental Analysis and Ftir Imaging for Breast Tissue Analysis
Source: Sci Rep. 2019 Jul 8;9:9854. doi: 10.1038/s41598-019-46056-4 (PMC6614471; doi:10.1038/s41598-019-46056-4)
Supplement: Supplementary file 1 — S1 [file 41598_2019_46056_MOESM1_ESM.pdf]

AN INNOVATIVE PLATFORM MERGING ELEMENTAL ANALYSIS AND FTIR IMAGING FOR BREAST TISSUE ANALYSIS

Mohamed H. Ali[1], Fazle Rakib[2], Khalid Al-Saad[2], Rafif Al-Saady[3] and Erik Goormaghtigh[4]

[1]Qatar Biomedical Research Institute (QBRI), HBKU, Doha-Qatar.
[2]Department of Chemistry and Earth Sciences, Qatar University, Doha-Qatar.
[3]Pathology and Laboratory Medicine, Al Ahli Hospital, Doha-Qatar,
[4]Center for Structural Biology and Bioinformatics, Laboratory for the Structure and Function of Biological Membranes, Campus Plaine CP206/02; Université Libre de Bruxelles CP206/2, B1050 Brussels, Belgium

Supplementary Materials

Figure S1 displays the distribution of 9 elements in 6 breast tissue sections.

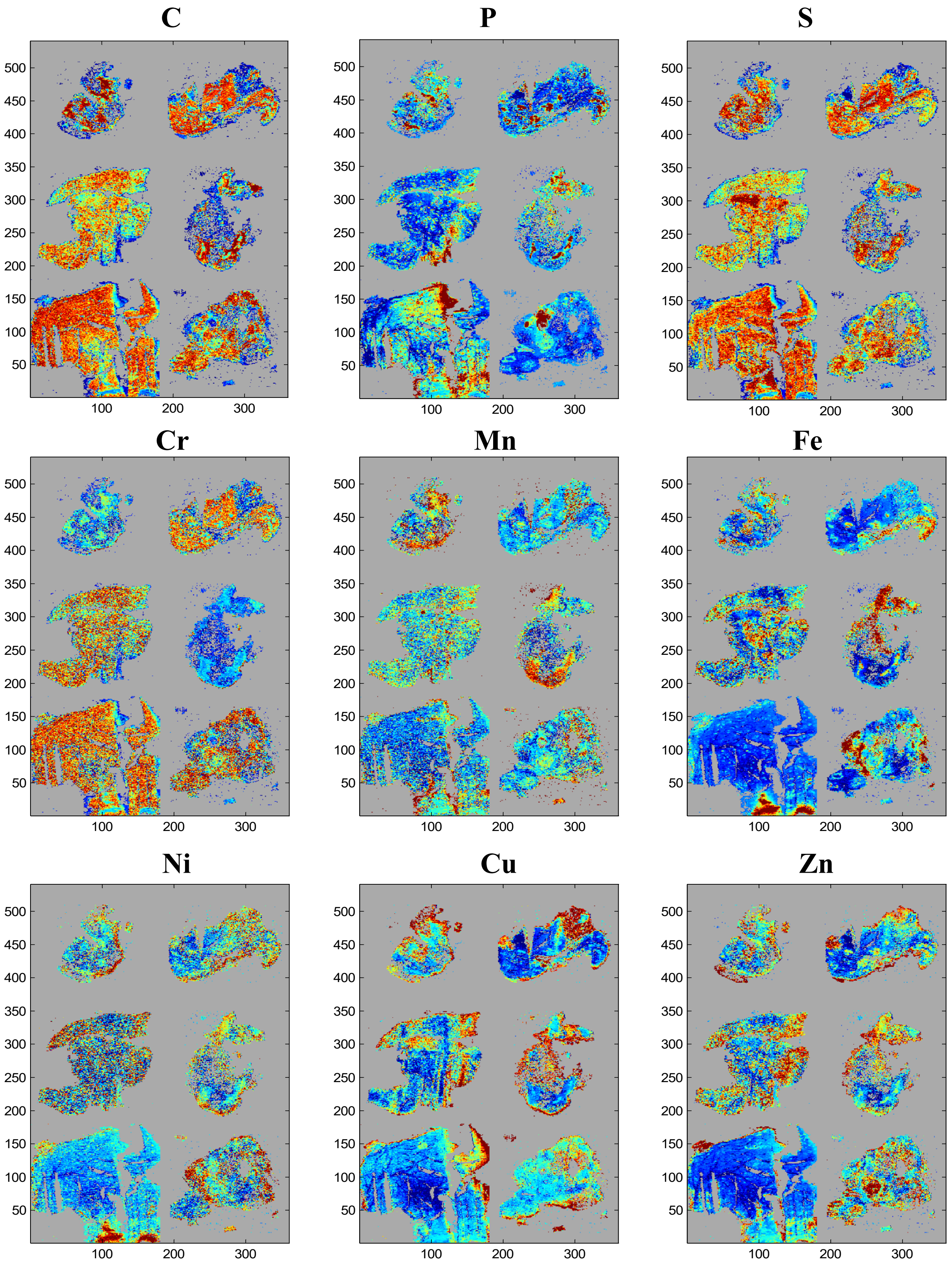

Figure S1: distribution of ^{13}C, ^{31}P, ^{34}S, ^{52}Cr, ^{55}Mn, ^{56}Fe, ^{58}Ni, ^{63}Cu and ^{64}Zn *for 6 breast tissue sections. Using the* ^{13}*C map, values below -0.2 were filtered out and displayed in grey. The rest of the scale between blue and red cover a percentile 95 of the values. The remaining 5% of the values were set at the minimum or maximum values found for 95% of the values*
